# Supplementary material for: The Impact of Body Mass Composition on Outcome in Multiple Traumatized Patients—Results from the Fourth Thoracic and Third Lumbar Vertebrae: A Single-Center Retrospective Observational Study
Source: J Clin Med. 2023 Mar 27;12(7):2520. doi: 10.3390/jcm12072520 (PMC10095228; doi:10.3390/jcm12072520)
Supplement: Supplementary file 1 [file jcm-12-02520-s001.zip › jcm-2149107-supplementary.pdf]

Table S1. Correlations between BC variables and clinical course.

Body composition		L3 SMRA	L3 VATI	L3 SMI	L3 SATI	T4 SMRA	T4 SMI	T4 SATI	age
**Hospital stay**	**Pearson**	0.025	0.025	0.106	0.115	0.054	0.022	**0.128**	-0.087
	**p**	0.674	0.672	0.074	0.052	0.360	0.711	**0.030**	0.136
**ICU stay**	**Pearson**	-0.024	0.042	0.070	0.106	-0.005	-0.015	**0.127**	-0.024
	**p**	0.686	0.478	0.238	0.074	0.936	0.803	**0.032**	0.677
**Duration of ventilation**	**Pearson**	-0.074	0.073	0.020	0.112	-0.048	-0.051	**0.151**	0.040
	**p**	0.212	0.222	0.738	0.059	0.424	0.396	**0.011**	0.494
**CCI**	**Pearson**	-0.093	0.032	-0.014	-0.048	-0.102	0.060	-0.015	**0.136**
	**p**	0.116	0.585	0.812	0.420	0.085	0.310	0.805	**0.019**
**ISS**	**Pearson**	0.030	-0.028	-0.037	-0.049	-0.004	-0.008	-0.009	-0.040
	**p**	0.616	0.635	0.529	0.406	0.948	0.892	0.883	0.492
**Death**	**Eta coefficient**	**0.208**	0.100	**0.151**	0.062	**0.165**	0.084	0.014	**0.268**
	**p**	**0.000**	0.091	**0.011**	0.294	**0.005**	0.157	0.817	**0.000**
**Age**	**Pearson**	**-0.667**	**0.560**	**-0.252**	**0.170**	**-0.576**	**-0.240**	**0.398**	
	**p**	**0.000**	**0.000**	**0.000**	**0.004**	**0.000**	**0.000**	**0.000**	

ICU: intensive care unit; CCI: Comprehensive Complication Index; ISS: Injury Severity Score; SMRA: Skeletal muscle radiation attenuation; VATI: Visceral adipose tissue index; SMI: Skeletal muscle index; SATI: Subcutaneous adipose tissue index.

Table S2. Correlations of BC parameters and local and systemic complications.

Body composition		L3 SMRA	L3 VATI	L3 SMI	L3 SATI	T4 SMRA	T4 SMI	T4 SATI	Age
**Occurrence of overall complications**	**Eta-coefficient**	0.046	0.046	0.089	0.017	0.072	0.079	0.069	0.055
	**p**	0.440	0.439	0.135	0.778	0.225	0.183	0.246	0.344
**Local complications**	**Eta-coefficient**	0.062	0.020	0.065	0.042	0.028	**0.130**	0.066	0.036
	**p**	0.298	0.734	0.278	0.478	0.638	**0.029**	0.267	0.541
**Systemic complications**	**Eta-coefficient**	**0.140**	0.049	**0.182**	**0.136**	0.104	**0.156**	**0.148**	**0.115**
	**p**	**0.018**	0.408	**0.002**	**0.021**	0.080	**0.008**	**0.013**	**0.048**

Body composition		L3 SMRA	L3 VATI	L3 SMI	L3 SATI	T4 SMRA	T4 SMI	T4 SATI	Age
Clavien-Dindo score	Kendall-tau	**-0.127**	**0.095**	**-0.102**	0.043	**-0.101**	**-0.108**	0.056	**0.185**
	p	**0.003**	**0.027**	**0.017**	0.313	**0.018**	**0.012**	0.190	**0.000**
	Spearman	**-0.180**	**0.130**	**-0.139**	0.057	**-0.143**	**-0.148**	0.077	**0.254**
	p	**0.002**	**0.028**	**0.019**	0.342	**0.015**	**0.012**	0.193	**0.000**

SMRA: Skeletal muscle radiation attenuation; VATI: Visceral adipose tissue index; SMI: Skeletal muscle index; SATI: Subcutaneous adipose tissue index.

Table S3. Regression models of BC parameters with regard to local and systemic complications.

Body composition			L3 SMRA	L3 VATI	L3 SMI	L3 SATI	T4 SMRA	T4 SMI	T4 SATI	Age
Occurrence of complications	univariable	Odds ratio	0.990	1.001	0.992	1.000	0.980	0.997	1.002	1.007
		95% CI	0.966 - 1.015	0.998 - 1.004	0.982 - 1.002	0.998 - 1.003	0.949 - 1.012	0.992 - 1.002	0.999 - 1.005	0.993 - 1.020
		p	0.439	0.438	0.136	0.777	0.225	0.183	0.246	0.343
	multi-variable	Odds ratio								
		95% CI								
		p								
Local complications	univariable	Odds ratio	0.989	1.000	0.995	1.001	0.993	**0.995**	1.002	1.004
		95% CI	0.967-1.010	0.997 - 1.002	0.986 - 1.004	0.998 - 1.003	0.65 - 1.022	**0.990 - 0.999**	0.999 - 1.004	0.992 - 1.016
		p	0.298	0.733	0.277	0.477	0.636	**0.030**	0.267	0.539
	multi-variable	Odds ratio						**0.994**		
		95% CI						**0.989 - 0.999**		
		p						**0.025**		
	univariable	Odds ratio	**0.969**	1.001	**0.982**	**1.003**	0.967	**0.991**	**1.004**	1.015

Body composition			L3 SMRA	L3 VATI	L3 SMI	L3 SATI	T4 SMRA	T4 SMI	T4 SATI	Age
Systemic complications		95% CI	**0.943 - 0.995**	0.998 - 1.005	**0.971 - 0.994**	**1.000 - 1.006**	0.932 - 1.004	**0.984 - 0.998**	**1.001 - 1.008**	**1.000 - 1.030**
		p	**0.020**	0.407	**0.003**	**0.024**	0.081	**0.009**	**0.014**	**0.050**
	multi-variable	Odds ratio	0.975		**0.983**	**1.003**		**0.991**	**1.004**	1.014
		95% CI	0.940 - 1.011		**0.972 - 0.996**	**1.000 - 1.006**		**0.984 - 0.998**	**1.000 - 1.008**	0.998 - 1.029
		p	0.167		**0.007**	**0.027**		**0.012**	**0.036**	0.078
Clavien-Dindo score	univariable	95% CI	**(-0.069) - (-0.020)**	**0.000 - 0.006**	**(-0.024) - (-0.004)**	(-0.002) - 0.003	**(-0.074) - (-0.008)**	**(-0.011) - (-0.001)**	(-0.002) - 0.005	**1.225 - 2.609**
		p	**0.000**	**0.048**	**0.009**	0.809	**0.014**	**0.027**	0.358	**0.000**
	multi-variable	95% CI	(-0.031) - 0.019	(-0.003) - 0.002	**(-0.016) - (-0.016)**		(-0.017) - 0.042	**(-0.010) - (-0.002)**		**0.013 - 0.033**
		p	0.630	0.697	**0.029**		0.420	**0.004**		**0.000**

SMRA: Skeletal muscle radiation attenuation; VATI: Visceral adipose tissue index; SMI: Skeletal muscle index; SATI: Subcutaneous adipose tissue index.

Table S4. Univariate and multivariate analysis of BC variables and clinical course.

Body composition			L3 SMRA	L3 VATI	L3 SMI	L3 SATI	T4 SMRA	T4 SMI	T4 SATI
Hospital stay (d)	univariable	95% CI	(-0.220) - 0.341	(-0.028) - 0.043	(-0.028) - 0.043	0.000 - 0.061	(-0.196) - 0.538	(-0.047) - 0.069	**0.004 - 0.077**
		p	0.674	0.672	0.074	0.052	0.360	0.711	**0.030**
	multi-variable	95% CI							**0.023 - 0.102**
		p							**0.002**
ICU stay (d)	univariable	95% CI	(-0.306) - 0.202	(-0.020) - 0.044	(-0.041) - 0.166	(-0.003) - 0.053	(-0.345) - 0.317	(-0.059) - 0.046	**0.003 - 0.069**
		p	0.686	0.478	0.238	0.074	0.936	0.803	**0.032**
	multi-variable	95% CI							**0.015 - 0.085**
		p							**0.006**
Duration of ventilation (h)	univariable	95% CI	(-0.324) - 0.072	(-0.009) - 0.041	(-0.068) - 0.095	(-0.001) - 0.043	(-0.364) - 0.154	(-0.059) - 0.023	**0.008 - 0.059**

Body composition			L3 SMRA	L3 VATI	L3 SMI	L3 SATI	T4 SMRA	T4 SMI	T4 SATI
		p	0.212	0.222	0.738	0.059	0.424	0.396	**0.011**
	multi-variable	95% CI							**0.295 - 1.612**
		p							**0.005**
CCI	univariable	95% CI	(-0.989) - 0.109	(-0.050) - 0.089	(-0.253) - 0.198	(-0.085) - 0.036	(-1.349) - 0.088	(-0.055) - 0.174	(-0.082) - 0.063
		p	0.116	0.585	0.812	0.420	0.085	0.310	0.805
	multi-variable	95% CI							
		p							
ISS	univariable	95% CI	(-0.066) - 0.112	(-0.014) - 0.009	(-0.048) - 0.025	(-0.014) - 0.006	(-0.121) - 0.113	(-0.020) - 0.017	(-0.013) - 0.011
		p	0.616	0.635	0.529	0.406	0.948	0.892	0.883
	multi-variable	95% CI							
		p							

ICU: intensive care unit; CCI: Comprehensive Complication Index; ISS: Injury Severity Score; SMRA: Skeletal muscle radiation attenuation; VATI: Visceral adipose tissue index; SMI: Skeletal muscle index; SATI: Subcutaneous adipose tissue index.
